# Selection favors loss of floral pigmentation in a highly selfing morning glory

**DOI:** 10.1371/journal.pone.0231263

**Published:** 2020-04-13

**Authors:** Tanya M. Duncan, Mark D. Rausher

**Affiliations:** Department of Biology, Duke University, Durham, NC, United States of America; Zhejiang University, CHINA

## Abstract

A common evolutionary trend in highly selfing plants is the evolution of the “selfing syndrome”, in which traits associated with pollinator attraction are lost or greatly reduced. Limited information is available on whether these trait reductions are favored by natural selection or result from reduced purifying selection coupled with genetic drift. This study attempted to distinguish between these two possibilities for the evolutionary loss of floral pigmentation in the highly selfing species *Ipomoea lacunosa*. This study also tested the hypothesis that loss of floral pigmentation is caused by downregulation or loss of function in a tissue-specific anthocyanin transcription factor, as has been found in other plants. F2 individuals of a cross between white and pigmented individuals revealed segregation at two epistatically acting loci: one affecting pigmentation in both corolla throat and limbs (*Anl1*) and one affecting limb pigmentation (*Anl2*). Individuals that are homozygous for the “white” allele at *Anl1* have white throats and limbs regardless of genotype at *Anl2*. In individuals with pigmented throats, homozygosity of the “white” allele at *Anl2* produces white limbs. Flower color variation at *Anl1* cosegregates with an *R2R3-Myb* anthocyanin transcription factor, which is down-regulated in white-flowers but not in pigmented flowers. Differential expression of the two alleles of this gene indicates that down regulation is caused by a *cis*-regulatory change. Finally, allele-frequency differences at *Anl1* were substantially and significantly greater than differences in allele frequencies at four microsatellite loci. These results are consistent with the hypotheses that the identified *R2R3-Myb* gene corresponds to *Anl1* and that evolutionary loss of pigmentation in *I*. *lacunosa* was caused by selection. They are also consistent with previous studies demonstrating that loss of floral pigmentation is usually caused by down-regulation or functional inactivation of an *R2R3-Myb* gene.

## Introduction

The evolution of selfing from outcrossing is one of the most frequent mating system transitions in angiosperms and has occurred in most plant families [1,2]. Change in a suite of floral traits typically accompanies this transition, giving rise to a characteristic “selfing syndrome”, in which many floral traits are substantially reduced [1,3]. These traits may be divided into two categories. First, there are traits that directly contribute to an increase in the rate and/or efficiency of selfing (“causal” traits). This category includes traits such as self-compatibility and reduced physical and temporal separation of anthers and stigma. When selfing is favored, selection is expected to operate to favor these traits. The second category of traits is those that do not necessarily contribute directly to enhanced selfing (“ancillary” traits). These traits are typically associated with reduced pollinator attraction and include decreased pollen/ovule ratios, reduced flower size, nectar and scent production, and sometimes loss of pigmentation. In the early stages of the evolution of increased selfing, selection may favor these traits because they increase selfing by reducing visitation by pollinators. Once a high selfing rate has evolved, selection may favor reallocation of resources away from these traits to other traits that enhance fitness [4]. However, it is also possible that these traits degenerate simply because they accumulate mutations that are not opposed by purifying selection because pollinator attraction is no longer needed. Such degeneration would occur by genetic drift and is not necessarily adaptive.

The relative involvement of selection and drift in the evolution of ancillary traits contributing to the selfing syndrome has seldom been examined (but see [5–7]). One of the objectives of this study was to determine whether selection has contributed to the evolution of loss of pigmentation in a highly selfing species of morning glory, *Ipomoea lacunosa*.

In general, anthocyanins are produced in angiosperms by a series of enzymes that are activated by a transcription factor complex composed of a Group 6 *R2R3-Myb* protein, a *bHLH* protein, and a *WDR40* protein [8–10]. The latter two proteins typically also regulate processes in addition to anthocyanin production, including production of proanthocyanidins, vacuolar acidification, and the production of trichomes and root hairs [8–10]. By contrast, the *R2R3-Myb* proteins are specific to anthocyanin production, and often different copies regulate pigmentation in different tissues and in different parts of the flower [11,12]. Because of this specificity, down-regulation or loss of function of a specific *R2R3-Myb* gene is expected to have substantially fewer deleterious fitness effects than similar changes in either the *bHLH* or *WDR40* genes.

Loss of floral pigmentation, particularly loss of anthocyanins, is a common evolutionary transition in plants. Elimination of anthocyanins can be achieved by several distinct types of mutation: (1) loss-of-function mutations in enzyme-coding genes of the anthocyanin pathways; (2) cis-regulatory mutations causing marked downregulation in enzyme-coding genes; (3) loss-of-function mutations in transcription factors that activate pathway enzyme-coding genes; or (4) mutations that cause downregulation of such transcription factors [13]. All of these types of mutation are known from investigations of horticultural variants, and thus presumably could be targeted by selection to eliminate floral anthocyanins [13]. However, it has been suggested that not all of these types of mutation are equally likely to contribute to evolutionary loss of pigmentation because they differ in their pleiotropic effects. They therefore are expected to differ in their net selective advantage, which determines the probability that they will become fixed substitutions [13].

In particular, it is expected that mutations of type (1) will have large adverse deleterious pleiotropic effects because enzyme-coding genes are generally expressed in many tissues besides flowers, and they produce many flavonoids that are of ecological or physiological importance to the plant [9, 14–16]. Mutations of type (3) are likely to have similar adverse effects if the transcription factor is also broadly expressed in many tissues or affects many characters, as is true typically true for the transcription factors *bHLH* and *WDR40*, but not for *R2R3-Mybs*. By contrast, mutations of type (2) and (4) are likely to have few pleiotropic effects, especially if mutation causes only flower-specific downregulation. In keeping with these expectations, almost all examined cases of evolutionary pigment loss in flowers are due to either downregulation of, or loss of function in, floral-specific *R2R3 Myb* transcription factors that activate the anthocyanin pathway [12,13, 17–19]. A second objective of this study was to evaluate whether the mutation (s) responsible for loss of pigments in *I*. *lacunosa* also conforms to this pattern.

## Materials and methods

### Statement of permit requirements

No permits were required for this study.

### Study organisms

**Fig 1:**
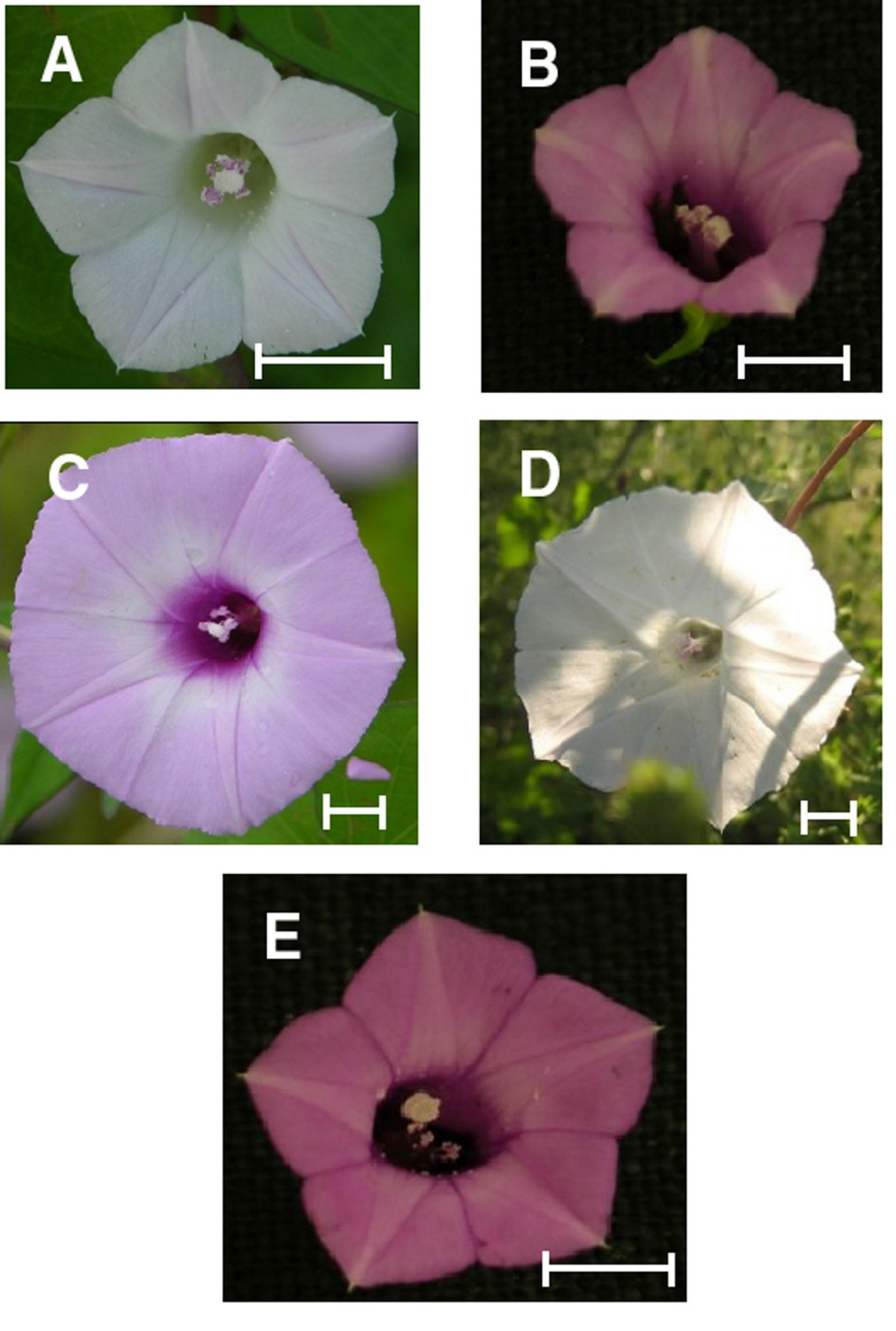
Flowers of species used in this study. A:white *Ipomoea lacunosa*. B: purple *I*. *lacunosa*. C: purple *I*. *cordatotriloba*. D: white *I*. *cordatotriloba*. E: *I*. ⨯ *leucantha*. Scale bar indicates 5 mm.

*Ipomoea lacunosa* and *Ipomoea cordatotriloba* (Convolvulaceae) are noxious weeds that are indigenous to the southeastern United States [20]. The two plants have different floral morphologies, with *Ipomoea lacunosa* typically having smaller white flowers, while *I*. *cordatotriloba* typically has larger, purple flowers [21] (Fig 1). Although both taxa have a characteristic flower color, white-flowered *I*. *cordatotriloba* and purple-flowered *I*. *lacunosa* individuals can be found in nature. A recent phylogenetic analysis of the *Batatas* section of *Ipomoea* indicates that these are sister species [22]. The two species are partially reproductively isolated by a crossing barrier, although hybrids can be obtained at low frequencies, and extensive one-way gene flow from *I*. *lacunosa* to *I*. *cordatotriloba* has occurred in regions of sympatry [23]. In the states of North and South Carolina, *I*. *lacunosa* grows along the coast as well as in the central area of the two states, while *I*. *cordatotriloba* is found predominately along the coast. A third taxon, *I*. ⨯ *leucantha*, grows in the same region and is believed to be a stable hybrid formed by a cross between *I*. *lacunosa* and *I*. *cordatotriloba* [21]. In North and South Carolina the plants germinate in late May and begin to flower in August or early September. Flowering ceases sometime in mid to late fall, and plants die at the first hard frost. Plants of each taxon are self-compatible, with *I*. *lacunosa* being highly selfing and *I*. *cordatotriloba* having a mixed mating system [23,24].

### Identification of anthocyanidins using HPLC

Anthocyanidins, the breakdown products of anthocyanin acid hydrolization, were extracted and identified with high-performance liquid chromatography, using a previously described method [25] in the stem and flowers of 3 purple- and 3 white- flowered *I*. *lacunosa* as well as 2 purple flowered *I*. *cordatotriloba*.

### Genetics of flower color

Ten white- and 10 purple-flowered *I*. *lacunosa* individuals were used to create 10 F_1_ hybrids. Each hybrid was allowed to self to produce S_2_ individuals. Approximately 75 S_2_ seeds from each line were grown 1 meter apart in a field plot at Duke University. Flower color (purple vs. white) of the inner (tube) and outer (limb) corolla were recorded on each plant on three different occasions.

In a second experiment, F_1_ hybrids were created by crossing 4 purple and 4 white flowered *I*. *lacunosa* parental plants in all pairwise combinations. A total of 140 S_2_ progeny were grown in a greenhouse at Duke University. The outer corolla was phenotyped as light-, medium-, or dark-, and photographs were taken to ensure accuracy of results.

### Identification of anthocyanin genes

We cloned several genes from the anthocyanin biosynthetic pathway. Partial coding sequences for genes for the enzymes dihydroflavonol reductase (*IlacDfr*) and chalcone synthase (*IlacChs*) as well as for an *R2R3-Myb* anthocyanin transcription factors known to control their expression in the flowers of *Ipomoea* [26–28] were amplified from *I*. *lacunosa* floral mRNA, using primers developed from relevant sequences in other species of *Ipomoea* (S1 Table). RNA was extracted using the SIGMA, Spectrum Total RNA extraction kit, and cDNA was produced using Invitrogen M-MLV reverse transcriptase. Each amplified sequence was Blasted against the National Center of Biotechnology Information protein database to confirm that it showed highest similarity to previously identified anthocyanin genes. Additionally, 345 bp of the 3'UTR region of the *R2R3-Myb* gene was PCR amplified out of DNA of *I*. *lacunosa* and *I*. ⨯ *leucantha* using primers developed from *I*. *nil* (S1 Table). Sequences of all genes are available from Genbank (accession numbers MT161448– MT161455).

### Quantifying gene expression levels

We used quantitative real-time PCR (qPCR) to compare relative expression levels of the anthocyanin genes between species and among S_2_ individuals. Additionally, qPCR was conducted on 4 purple and 4 white *I*. *lacunosa* that had been field collected as seed and grown in the greenhouse. Flower buds were collected between 4:00 PM– 5:00 PM the day before anthesis in an attempt to standardize the developmental stage in which the flower tissue was collected. A 0.5 cm-long section of the inner corolla (throat) was flash-frozen in liquid nitrogen and stored at -80°C until RNA was extracted as described above.

For qPCR reactions, 300 ng of RNA were used to make 20 μl of cDNA for each sample, using methods described above. One microliter of cDNA was then used in a 20 μl SYBR green qPCR reaction. The reaction mix included 10 μl Dynamo SYBR green qPCR mix (REF), 0.2 μl primers, 0.4 μl Rox Passive Dye. Reactions were run on an ABI Prism 7000 Sequence Detection System using the following cycling protocol: 94°C for 10 minutes, 40 cycles of 94°C for 20 seconds, 55°C for 30s, and 72°C for 45 seconds. Q-PCR primers were designed to yield a product no larger than 200 bp (S2 Table). Relative expression of the target gene was calculated using the method developed by Peirson et al. 2003 [29]. One purple-flowered plant was designated as the control to which all other expression levels were compared, and an ANOVA was used to determine whether there was a significant difference in expression of anthocyanin genes between purple and white individuals. All ANOVAs were conducted using JMP®, Version 9. SAS Institute Inc., Cary, NC, 1989–2007.

### Co-segregation analyses

We examined whether any of the anthocyanin genes we had identified co-segregated with flower color. Because we were unable to find genetic markers to differentiate anthocyanin genes from purple and white *I*. *lacunosa*, we created 3 F_1_ individuals by crossing white-flowered *I*. *lacunosa* to its close relative *I*. ⨯ *leucantha*, which has purple flowers and scorable allelic differences. The F_1_ plants produced very few seeds when allowed to self. Therefore, 41 B_2_ seeds were generated by backcrossing the F_1_ plants to white flowered *I*. *lacunosa*. Additionally, one purple flowered F_2_ plant was backcrossed to white *I*. *lacunosa* and 12 F_3_ plants were created.

DNA was extracted from the three parental *I*. ⨯ *leucantha* and *I*. *lacunosa*, as well as from 3 F_1_ and their F_2_ and F_3_ backcrossed progeny using a Cetyl Trimethyl Ammonium Bromide (CTAB) protocol [30]. Genotypes of F2 progeny were determined by cutting PCR fragments of *IlacDfr* and *R2R3-Myb* with NdeI and AseI, respectively, since an NdeI restriction site is present in the *I*. *lacunosa* sequence but not in the *I*. *⨯ leucantha* sequence (S4D Fig), and an AseI restriction site is present in the *I*. *lacunosa* sequence but not in the *I*. ⨯ *leucantha* sequence (S4E Fig). (S3 Table).

### Allele-specific expression

The above analyses revealed that the *R2R3-Myb* gene is down-regulated in white-flowered individuals. To determine if this downregulation was due to a *cis-* or a *trans-*regulatory change, we quantified allele-specific expression levels in heterozygotes [31]. The alleles are differentiated by a G/T polymorphism in the third exon of the gene (S1 Fig). RNA was extracted from throat tissue and cDNA was generated using methods described above. PCR amplification was performed by pyrosequencing on four cDNA and four genomic DNA replicates for each of three F_1_ individuals as well as non-template and non-sequencing primer controls. Pyrosequencing reactions used PyroMARKTMQ961D (Qiagen) [31,32]. The DNA analysis provides a control for inherent differences in production of the two alleles by PCR.

Allele expression has been shown to be directly correlated to the peak sequencing height generated in a pyrosequencing reaction [31]. An ANOVA was used to detect if the purple and white allele showed significantly different proportional expression of the two alleles in cDNA and genomic DNA.

### Flower color census

In the fall of 2010, a large population census was conducted of *I*. *cordatotriloba and I*. *lacunosa*. During the census, flower color frequency was taken on 50 populations of *I*. *cordatotriloba* growing in both North and South Carolina and 43 populations of *I*. *lacunosa* in North Carolina (S4 Table). To measure the flower color frequency in a population, two transects were taken. Plants were sampled at 2 m intervals along transects to ensure that different individuals were scored. When flower color was fixed in the population, as determined by an initial visual inspection, we scored 100 flowers. However, when there was obvious variation in flower color in a population, 200 flowers were scored.

### Microsatellite identifying and scoring

DNA was extracted using a CTAB protocol [30] using primers that had been developed for *I*. *trifidia* but were also reported to amplify microsatellite regions in *I*. *lacunosa* [33]. Out of the 8 microsatellites reported to amplify in *I*. *lacunosa*, we found that only 4 amplified and contained sufficient variability to distinguish among the study taxa (S4 Table). Each of the 4 microsatellite markers was amplified with Hex or Fam fluorescently labeled primers, using KAPA taq (Kapa Biosystems, Woburn, Massachusetts, USA), and fragment analysis was conducted on a ABI 3730 x 1 DNA Analyzer. Each marker was visually scored using the software GENEMARKER (SoftGenetics, 2005, State College, Pennsylvania, USA).

### Frequencies of flower color versus neutral genetic loci

To determine whether divergence in flower color between *I*. *lacunosa* and *I*. *cordatotriloba* is consistent with neutral expectations, we conducted a bootstrap analysis to compare allele frequency differences between the two species at the flower-color locus with differences in frequencies at the microsatellite loci. Because the census data did not permit us to determine whether purple-flowered individuals are homozygous or heterozygous, we estimated allele frequency in two ways. First, we assumed that there were no heterozygotes and that all purple individuals were therefore homozygous (Method 1). The frequency of the white allele in this case is estimated as the frequency of white individuals in a population. Alternatively, we assumed that genotype frequencies at the flower-color locus are in Hardy-Weinberg equilibrium (Method 2). In this case, the frequency of the white allele is estimated as the square root of the proportion of white-flowered individuals. These two cases represent extreme possibilities that bracket the true proportions of heterozygotes in the population.

For the bootstrap analysis, we first generated for the microsatellite markers a distribution of between-species average difference in allele frequency, where the average was taken over loci. Each bootstrap sample was obtained in the following way: first populations were randomly sampled with replacement within a species. Within each sampled population, individuals were chosen randomly with replacement. Once the sample had been reconstituted in this way, loci were randomly sampled with replacement. Average difference in allele frequency was then calculated as follows: first the population allele frequencies were calculated for each locus. These were then averaged to obtain an average frequency of each locus in each species. The absolute values of the differences in frequency between species were then calculated for each locus, and these were averaged over loci to obtain a final value.

Bootstrap samples for allele frequencies at the flower-color locus were calculated in similar fashion. We started with a data set that contained either 100 or 200 individuals in each population, depending on the number of individuals sampled. Genotypes for these individuals were assigned based on the censused frequency of white alleles using either Method 1 or Method 2 (see above). For each bootstrap sample, populations were randomly sampled with replacement within species, and individuals were then randomly sampled with replacement for each sampled population. Population allele frequencies were averaged for each species, and the difference in allele frequency between species was calculated as the difference in population averages. One thousand bootstrap samples were used in the analysis.

### Data availability

Data for analyses in this study are available at Dryad under the unique identifier doi:10.5061/dryad.zcrjdfn7c

## Results

### Anthocyanidin production

While the anthocyanidins cyanidin and peonidin were detected in large and roughly equal amounts, as well as trace amounts of pelargonidin, in purple-flowered *I*. *lacunosa* and *I*. *cordatotriloba* individuals, they were not detectable in white-flowered individuals of *I*. *lacunosa* (Table 1 and S2 Fig), confirming that white flowers result from a lack of anthocyanin production. Anthocyanidins were detected in the stem of white flowered *I*. *lacunosa* (Table 1 and S2 Fig), indicating that anthocyanin pathway genes are functional in both species.

**Table 1:** Presence vs. absence of anthocyanins in different tissues for different species/flower color combinations.

Species	N	Corolla	Stem
*Ipomoea cordatotriloba*	2	Yes	Yes
*I*. *lacunosa* (purple- flowered individual)	3	Yes	Yes
*I*. *lacunosa* (white-flowered individual)	3	No	Yes

“Yes” indicates anthocyanidins detected. “No” means not detected.

### Flower color genetics

Crosses between white and purple flowered *I*. *lacunosa* from a population in North Carolina indicate that flower color variation is controlled by variation at two loci with major effects that interact epistatically. Three phenotypes were evident in the S_2_ progeny scored: (i) individuals with purple pigment in both the throat and corolla limb; (ii) individuals with white corolla limbs with rays of purple and purple throats; and (iii) individuals with white throats and little or no pigment in the corolla limbs (S3 Fig). Out of 732 S_2_ progeny created from ten purple and white parental crosses, 530 individuals had a purple throat, while 202 had a white throat. These numbers are consistent with the hypothesis that pigmentation in the throat of *I*. *lacunosa* is controlled by a single dominant Mendelian locus, as indicated by lack of deviation from the expected 3:1 ratio of purple to white (pooled G-value = 2.57, df = 1, p = 0.11). We designate this locus *Anl1* (*Anthocyaninless 1*).

The gene controlling the corolla limb color acts epistatically with the gene controlling the throat of the flower. If the throat is white, then there is little pigment in the limb, and the effect of the locus on limb pigmentation is too small to be quantified. On the other hand, if the throat is purple, then variation in anthocyanin production in the petal limb is evident. Among the S_2_ individuals with purple throats there were 22 with light-, 54 with medium-, and 27 with dark-purple limbs, indicating that anthocyanins in the limbs are likely controlled by a co-dominant Mendelian locus with a 1:2:1 ratio of light-, medium-, and dark-purple flower colors, respectively (pooled G-value = 0.74, d.f. = 2, p = 0.69). An additional 30 S_3_ individuals were scored for other purposes, and their flower color was consistent with expectations of the single-locus model (Table 2). We designate this locus *Anl2* (*Anthocyaninless 2*). The remainder of this study focuses on the gene controlling throat color (*Anl1*), since the white genotype at this locus lacks pigment throughout the flower.

**Table 2:** Phenotypes of S_3_ individuals.

	Number of S_3_ Offspring			
Parental Genotype (phenotype)	aa_BB (IC:W, OC: N/A)	A_bb (IC:P, OC:W)	A_Bb (IC: P, OC: LP)	A_BB (IC:P, OC:DP)
AaBb (IC = P, OC = LP)	7	2	5	1
AaBB (IC = P, OC = DP)	0	0	0	7
aa_BB (IC = W, OC = N/A)	8	0	0	0

IC = inner corolla (throat); OC = outer corolla (limb); W = white; P = purple; LP = light purple. N/A: not applicable.

### Identification of anthocyanin pathway genes

We identified one copy each of the enzyme-coding genes *Chs* and *Dfr* (which we designate *IlacChs* and *IlacDfr*, respectively), as well as one *R2R3-Myb* gene (which we designate *IlacMyb1*) from petal RNA (S4 Fig). The sequence of the corresponding *R2R3-Myb* gene from purple-flowered *I*. *lacunosa* was identical to that of *IlacMyb1*. The sequences of these three genes are highly similar to sequences reported from *Ipomoea* species (S4A–S4C Fig). Partial sequences for *Dfr* and the *R2R3Myb* gene were also obtained from *I*. ⨯ *leucantha* (S4D and S4E Fig), and we designate these *IleuDfr* and *IleuMyb1*, respectively. Sequences from white-flowered and purple-flowered *I*. *lacunosa* were identical, while at least one SNP differentiated these sequences from those of *I*. ⨯ *leucantha* (S4 Fig).

### Gene expression level differences

**Fig 2:**
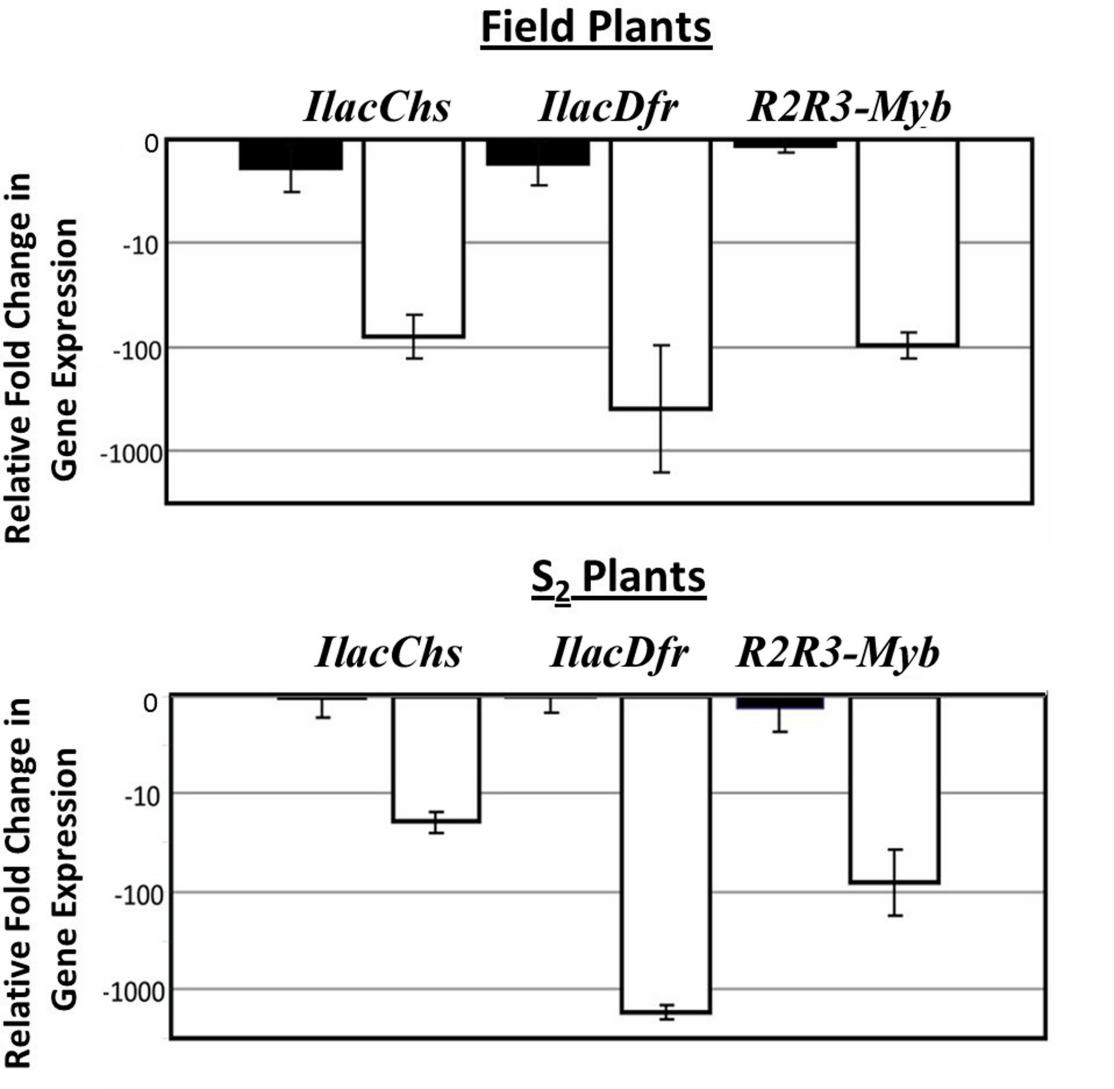
Expression levels of anthocyanin pathway genes in field and S_2_ individuals of *I*. *lacunosa*. Black bars are purple-flowered individuals, white bars are white-flowered individuals. Error bars indicate standard error. All relative fold changes are relative to a single purple-flowered *I*. *lacunosa*. Field individuals: *IlacChs* ANOVA, F_1,6_ = 93.80, p = 0.0002; *IlacDfr* ANOVA F_1,6_ = 39.63, p = 0.0015; *IlacMyb1* ANOVA, F_1,6_ = 533.95, p<0.0001; S_2_ individuals: *IlacChs* ANOVA, F_1,6_ = 52.14, p = 0.0007; *IlacDfr* ANOVA, F_1,6_ = 554.28, p<0.0001; *IlacMyb1* ANOVA, F_1,6_ = 31.44, p = 0.0025.

To determine whether white flowers were associated with altered regulation of anthocyanin pathway genes, we quantified transcript abundance for the enzyme-coding genes *IlacChs* and *IlacChsFR-B* and for the *IlacMyb1* transcription factor. We therefore examined expression levels of *IlacMyb1*. (We did not subsequently examine expression levels of *bHLH* or *WDR40* transcription factors because downregulation of *IlacMyb1* accounts for loss of pigmentation.) For flowers of field-collected *I*. *lacunosa* individuals, qPCR results revealed that *IlacChs* is almost 100-fold down-regulated (ANOVA, F_1,6_ = 93.80, p = 0.0002) and *IlacDfr* is more than 100- fold down-regulated (ANOVA F_1,6_ = 39.63, p = 0.0015) in the throats of white- compared to purple-flowered individuals (Fig 2). This coordinate downregulation suggests that the white phenotype is due to a genetic change in one of the transcription factors that regulates these anthocyanin genes. In keeping with this inference, qPCR showed that *IlacMyb1* is also down-regulated 100- fold (ANOVA, F_1,6_ = 533.95, p<0.0001) in white compared to purple field collected plants (Fig 2). Similarly, white S_2_ individuals, created from an original purple X white cross, show significant downregulation in all three genes compared to purple individuals (*IlacChs* ANOVA, F_1,6_ = 52.14, p = 0.0007; *IlacDfr* ANOVA, F_1,6_ = 554.28, p<0.0001; *IlacMyb1* ANOVA, F_1,6_ = 31.44, p = 0.0025) (Fig 2). These patterns suggest that downregulation of *IlacMyb1*is responsible for the production of white flowers.

### Co-segregation analyses

Co-segregation analysis supports the idea that the downregulation of *IlacMyb1* is responsible for the production of white flowers. Among 41 B_2_ offspring generated from backcrossing F_1_ (*I*. ⨯ *leucantha* crossed with *I*. *lacunosa*) individuals to white *I*. *lacunosa*, variation at *IlacMyb1* exhibited perfect association with variation in flower color (Table 3, families 1–3). Furthermore, the same association was found in 12 F_3_ offspring generated from a purple F_2_ backcrossed to white *I*. *lacunosa* (Table 3, family 4). By contrast, variation in *IlacDfr* did not co-segregate with flower color (Table 3). Family 4 was not included in the *IlacDfr* analysis because it did not contain an informative marker in the gene when backcrossed to white *I*. *lacunosa*. These results are consistent with the hypothesis that a *cis*-regulatory change in the *IlacMyb1* gene results in its own downregulation, as well as that of the two enzyme-coding genes.

**Table 3:** Co-segregation analysis of *R2R3-Myb* (*IlacMyb1* and *IleuMyb1*)and *Dfr* (*IlacDfr* and *IleuDfr*) with flower color.

			Flower Color			
Gene	Family	Genotype	purple	white	χ2	P
*R2R3-Myb*						
	1	*T*_	13	0	24	<0.001
		*tt*	0	11		
	2	*T*_	2	0	5	0.03
		*tt*	0	3		
	3	*T*_	5	0	12	<0.001
		*tt*	0	7		
	4	*T*_	3	0	12	<0.001
		*tt*	0	9		
	Total	*T*_	23	0		
		*tt*	0	30		
*Dfr*						
	1	*T*_	5	6	0.74	0.39
		*tt*	8	5		
	2	*T*_	1	1	0.33	0.57
		*tt*	1	2		
	3	*T*_	3	5	0.5	0.48
		*tt*	2	2		
	Total	*T_*	9	12		
		*Tt*	11	9		

(*T* = purple allele, *t* = white allele).

### Allele-specific expression

**Fig 3:**
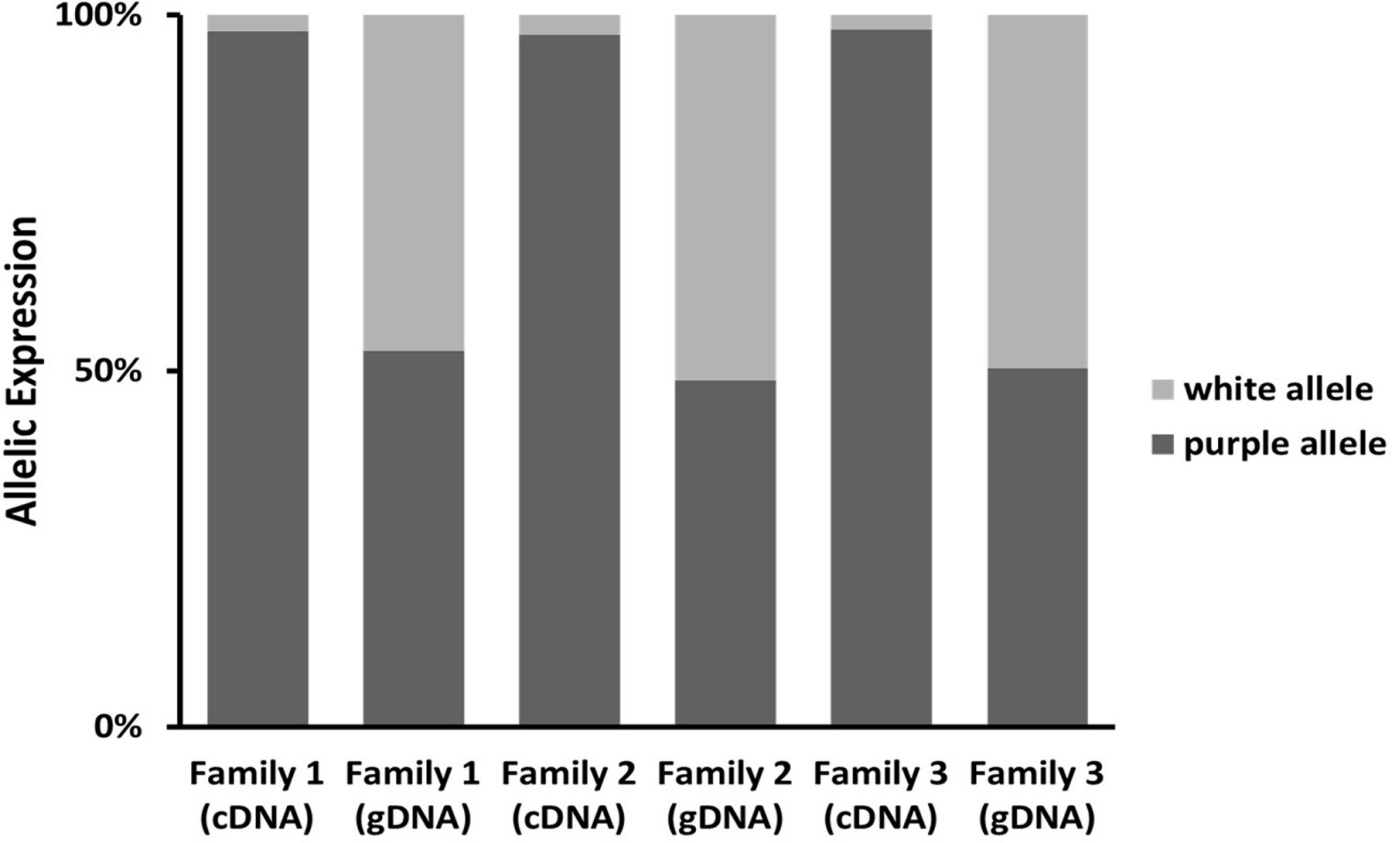
Allele-specific expression of *R2R3-Myb* in heterozygotes. Proportions of alleles sequenced using cDNA and gDNA as template for heterozygotes derived from three families.

To further test this hypothesis, we used allele-specific expression. If downregulation of the *Myb* gene is due to a *cis*-regulatory change, then in heterozygotes, the “white” allele should be expressed at much lower levels than the “purple” allele. By contrast, if a *trans*-acting regulatory is responsible, then the two alleles should be expressed at approximately the same level [31]. Allele-specific expression results obtained by pyrosequencing indicate that the downregulation of *IlacMy1b* in white flowers is due to a *cis* change (Fig 3). In F_1_ individuals created from a cross between white-flowered *I*. *lacunosa* and purple-flowered *I*. ⨯ *leucantha*, the “purple” allele was expressed at a significantly higher level in cDNA than the “white” allele. In all three replicates, the “purple” allele constituted more than 90% of the transcripts. By contrast, the two alleles exhibited equal amplification from the genomic DNA control. This difference between cDNA and gDNA is highly significant for each replicate: (Family 1: ANOVA, F_1,6_ = 443.36, p<0.0001; Family 2: F_1,6_ = 39.60, p = 0.0007; Family 3: F_1,6_ = 219.02, p<0.0001). This increased expression of the purple allele in F_1_ individuals indicates that the expression difference in the *R2R3*-*Myb* expression between white- and purple-flowered individuals is due to a *cis*-regulatory change. Combined with the co-segregation results, these differences in allele-specific expression strongly imply that *IlacMyb1* corresponds to *Anl1*.

### Test for neutral divergence in flower color

As expected, the average frequency of the white allele at *Anl1*, as reflected by the frequency of white-flowered individuals, differs between *I*. *lacunosa* and *I*. *cordatotriloba* (S5 Table). The average frequency for *I*. *lacunosa* was 0.98±0.004 (number of populations = 43), while that for *I*. *cordatotriloba* was 0.21±0.02 (number of populations = 50). This difference of 0.77 is highly significant (ANOVA F_1,91_ = 164.29, p<0.0001). By contrast, the average difference in microsatellite allele frequency between species was only 0.14, which is not significantly different. Only about 4% of the overall variation in allele frequencies is associated with differences between species (S6 Table).

**Fig 4:**
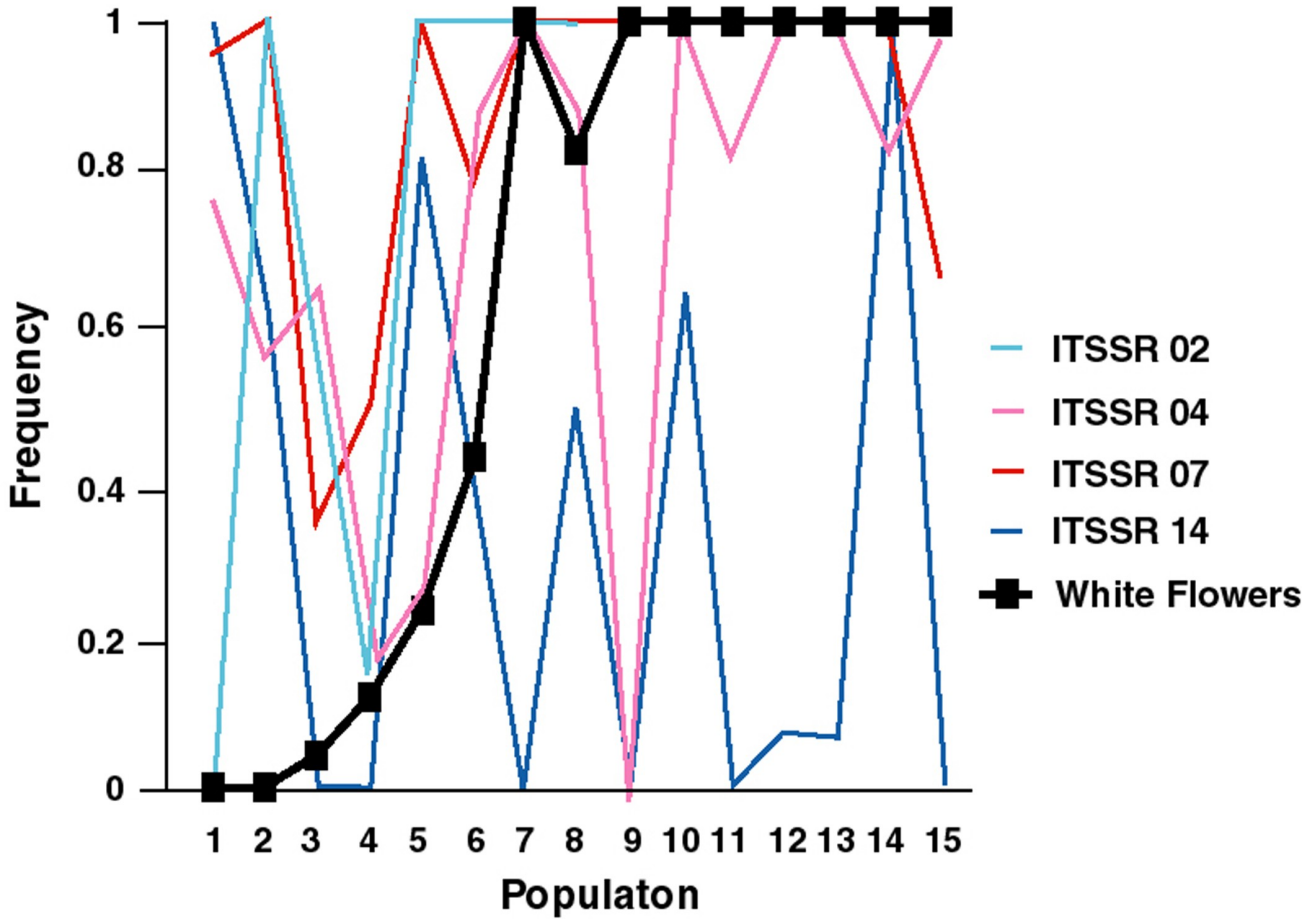
Variation in microsatellite allele frequencies and frequency of white flower across 15 populations with microsatellite data. Populations*1-7* contain *Ipomoea cordatotriloba* and 8–15 contain *I*. *lacunosa* individuals.

To determine whether the difference in these averages was statistically significant, we conducted a bootstrap analysis to generate a distribution of likely values for allele frequency differences. This analysis used a subset of the populations from the census because we had microsatellite data from only 8 *I*. *lacunosa* and 7 *I*. *cordatotriloba* populations (S7 Table and Fig 4). Nevertheless, these populations are representative of the larger sample of census populations for frequency of white flowers: The mean frequencies of white flowers for *I*. *lacunosa* and *I*. *cordatotriloba*, respectively were 0.978 and 0.259, which do not differ significantly from the proportions for samples not used (ANOVA on arcsin (square-root)-transformed data, F_1,47_ = 0.09, P = 0.76 and F_1,40_ = 0.01, P = 0.91 for *I*. *cordatotriloba* and *I*. *lacunosa*, respectively).

**Fig 5:**
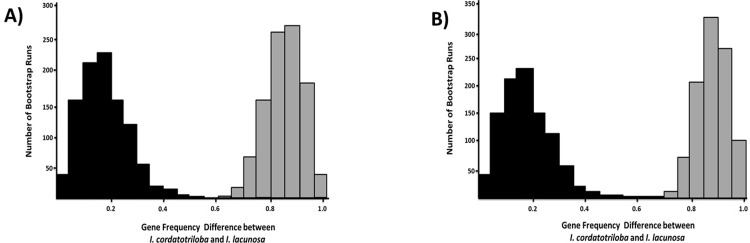
Bootstrapped between-species allele-frequency differences for microsatellite loci and *Anl1*. A) Analysis method 1. B) Analysis method 2. Black bars: Average allele frequency difference across microsatellite loci. Grey bars: Average allele frequency difference at *Anl1*.

Regardless of whether we used Method 1 or Method 2 to estimate *Anl1* allele frequencies, the bootstrap analysis gave essentially non-overlapping distributions for microsatellite frequencies and *Anl1* frequency differences between the two species (Fig 5). With Method 1, there was an overlap of one out of 1000 values, indicating that the two distributions differ at a significance level of P = 0.001. With Method 2, there was no overlap, indicating that the two distributions differ at a significance level of P<0.001. It thus seems that the magnitude of divergence at *Anl1* is not consistent with neutral divergence.

## Discussion

### Selective divergence at *Anl1*

One objective of this investigation was to determine whether evolutionary loss of pigmentation in *Ipomoea lacunosa* was a result of natural selection or genetic drift. Our finding that genetic differentiation between *I*. *lacunosa* and *I*. *cordatotriloba* at the *Anl1* locus is substantially and significantly greater than genetic differentiation at presumably neutral microsatellite loci (Fig 5) points to a role for selection in causing the evolution of white flowers in *I*. *lacunosa*. We believe this is a valid conclusion even though the number of neutral loci examined is small since the difference between allele frequency differences in microsatellite loci and at *Anl1* are highly significant.

There are several possibilities regarding the cause of this selection. One is that selection for increased selfing may promote the evolution of white flowers to reduce pollinator visitation and thereby increase selfing rate. This possibility is supported by previous studies on a related species, *Ipomoea purpurea*, which found that when white-flowered individuals were in the minority compared with purple-flowered individuals, white-flowered individuals were visited by pollinators less frequently and had an increased selfing rate relative to purple-flowered individuals [34–36]. This effect was frequency dependent, however, disappearing when white- and purple-flowered individuals were equally abundant. Alternatively, selection may have been generated by a cost of pigment production. The evolution of high selfing rates by other means (e.g., decreased anther-stigma separation) would likely have freed plants from dependence on pollinator visitation, thus eliminating the benefit of attracting pollinators. A cost of pigment production would then produce a net fitness disadvantage for pigmented flowers, leading to the fixation of the white allele at *Anl1*. Such costs have been demonstrated in other species [37], although they are not universal [38].

A final possible explanation for selection favoring the white allele in *I*. *lacunosa* is reinforcement, a process in which reduced fitness in hybrids selects for increased pre-zygotic reproductive isolation [39–42]. Under this explanation, gene flow from *I*. *cordatotriloba* to *I*. *lacunosa* would produce low-fitness hybrids, which would select for a reduction in between-species matings. Previous studies in other species of *Ipomoea* have demonstrated that pollinators exhibit non-random visitation in which visits are disproportionately between two white-flowered individuals or two purple-flowered individuals, with fewer than expected transitions between pigmented and white-flowered individuals [42], a pattern that would reduce gene flow between white- and purple-flowered individuals. It is thus possible that this process may have driven the evolution of white flowers in *I*. *lacunosa*. However, we view this scenario unlikely because gene flow between *I*. *lacunosa* and *I*. *cordatotriloba* is highly asymmetrical, with very little gene flow, if any, from *I*. *cordatotriloba* to *I*. *lacunosa* [23]. Nevertheless, we cannot definitively rule out this explanation because it is possible that gene flow was more extensive in the past. Distinguishing between these alternative explanations must await future investigations.

### Parallel genetic evolution

A question of current interest in evolutionary biology is the extent to which parallel phenotypic evolution is caused by parallel genetic or developmental evolution. Previous investigations of the evolutionary loss of floral pigmentation have revealed a remarkable degree of genetic parallelism: in all cases that have been examined, pigment loss has been caused by substitutions in floral *R2R3-Myb* genes [12, 17, 19]. This genetic parallelism presumably has occurred because, compared to other genes at which mutations can produce white flowers, inactivation or downregulation of the floral *Mybs* incurs relatively little deleterious pleiotropy because their normal expression domain is confined to flowers [13]. A second objective of this study was to determine whether loss of floral pigmentation in *I*. *lacunosa* conforms to this pattern.

Our results are consistent with this pattern. We have found that *IlacMyb1* appears to be down-regulated in *I*. *lacunosa* due to one or more cis-acting changes in its regulatory region(s) (Figs 2 and 3). We cannot rule out at this point that post-transcriptional regulatory changes may also be occurring. Additionally, we cannot definitively rule out that *IlacMyb1* also contains a loss-of-function mutation because we do not have a complete sequence of the gene from white- and purple-flowered individuals; however, only six codons of these genes are unsequenced, suggesting that this possibility has low probability. In either case, the observation that white-flowered *I*. *lacunosa* produce anthocyanins in their stems implies either that *IlacMyb1* is expressed in vegetative tissues and is functional, or that a different *R2R3Myb* gene is expressed. Thus, in either case, down-regulation of *IlacMyb1* only in flowers suggests that little deleterious pleiotropy is likely to be associated with this expression change.

Studies in other systems have also reported that cis-regulatory changes causing downregulation of anthocyanin *R2R3 Myb* genes have contributed to loss of anthocyanin pigmentation in flowers [43]. However, other studies have demonstrated that loss-of-function substitutions in the *R2R3 Myb* coding region have produced a similar evolutionary change [17]. These results thus suggest that both *cis*-regulatory and coding-region changes can cause parallel evolutionary change because they likely both have little to no adverse deleterious effects: both eliminate activation of the anthocyanin biosynthetic pathway in flowers but not in other tissues. There is thus not strict parallelism at the genetic level (i.e., the same mutation, nor even the same type of mutation, does not cause a similar phenotypic change), but at the developmental level: inactivation of the pigment pathway. A similar pattern has been found for evolutionary shifts from blue to red flowers, where inactivation of pathway branching enzymes causes a change in the type of anthocyanin produced, but this inactivation can be achieved by loss-of-function coding-region mutations, substitutions in *cis*-regulatory regions of the genes coding for these enzymes, or substitutions in transcription factors that activate them [44–46]. Thus, for different types of change in floral color, constraints giving rise to parallel phenotypic evolution appear to operate largely at the developmental level rather than at the genetic level.

## Supporting information

S1 Fig Sequence of portion of 3^rd^ exon of the *R2R3 Myb* corresponding to *Anl1*. Top sequence is for *I*. *lacunosa*. Bottom sequence is for *I*. *X leucantha*. Arrow designates single nucleotide polymorphism that differentiates the two sequences. Sequences of primers used for pyrosequencing are indicated.
(DOCX)

S2 Fig HPLC traces for standards, white-flowered *Ipomoea lacunosa* flowers, white-flowered *I*. *lacunosa* stems, purple-flowered *I*. *cordatotriloba* flowers, and purple-flowered *I*. *cordatotriloba* stems. (DOCX)

S3 Fig Three phenotypes of S2 offspring produced by selfing F1 individuals from a cross of a white-flowered *I*. *lacunosa* to a purple-flowered *I*. *lacunosa*. A. F1 individual. B. Individual with white throats and little or no pigment in the corolla limbs. C. Individual with white corolla limbs with rays of purple and purple throats. D. Individual with purple pigment in both the throat and corolla limb.
(DOCX)

S4 Fig Sequences of genes referred to in main text aligned with highly similar BLAST hits from *Ipomoea* species. - - - - - - - indicates indel; ●●●●●●● indicates not sequenced.
(DOCX)

S1 Table Primers used to amplify partial regions of anthocyanin genes. *DFR-B* and *CHS-D* as well as the anthocyanin transcription factor *R2R3-Myb*.
(DOCX)

S2 Table Primers used for Q-PCR expression assay. (DOCX)

S3 Table Primers and restriction enzymes used in co-segregation assay. (DOCX)

S4 Table Frequency of white allele in censused populations of *I*. *cordatotriloba* and *I*. *lacunosa*. 100 flowers were counted when populations were fixed for flower color. 200 flowers were counted when variation in flower color was found.
(DOCX)

S5 Table Microsatellite primers. Primers were developed and PCR optimized by Hu et al (Hu et al., 2004).
(DOCX)

S6 Table AMOVA for microsatellite variation. (DOCX)

S7 Table Flower color frequency for 15 populations with microsatellite data. (DOCX)
