# Supplementary material for: Selection favors loss of floral pigmentation in a highly selfing morning glory
Source: PLoS One. 2020 Apr 13;15(4):e0231263. doi: 10.1371/journal.pone.0231263 (PMC7153891; doi:10.1371/journal.pone.0231263)

Figure S1. Sequence of portion of 3^rd^ exon of the *R2R3 Myb* corresponding to *Anl1*. Top sequence is for *I. lacunosa.* Bottom sequence is for *I. X leucantha.* Arrow designates single nucleotide polymorphism that differentiates the two sequences. Sequences of primers used for pyrosequencing are indicated.


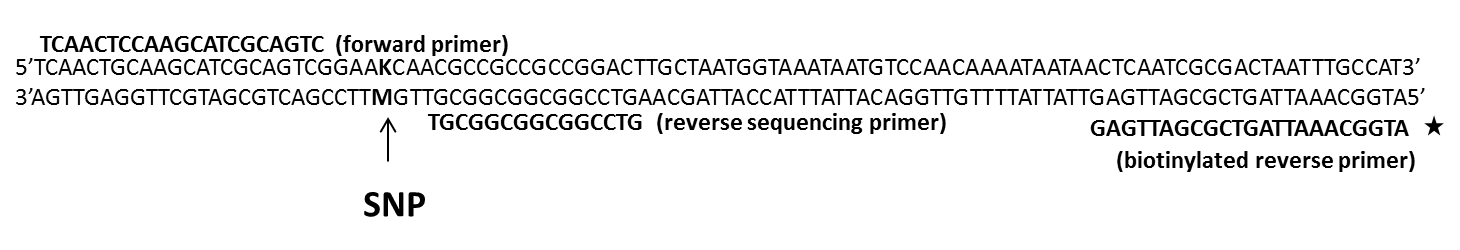

Supplement: S1 Fig — Top sequence is for I. lacunosa. Bottom sequence is for I. X leucantha. Arrow designates single nucleotide polymorphism that differentiates the two sequences. Sequences of primers used for pyrosequencing are indicated. (DOCX) [file pone.0231263.s001.docx]
