# Supplementary material for: Selection favors loss of floral pigmentation in a highly selfing morning glory
Source: PLoS One. 2020 Apr 13;15(4):e0231263. doi: 10.1371/journal.pone.0231263 (PMC7153891; doi:10.1371/journal.pone.0231263)

Figure S2. HPLC traces for standards, white-flowered *Ipomoea lacunosa* flowers, white-flowered *I. lacunosa* stems, purple-flowered *I. cordatotriloba* flowers, and purple-flowered *I. cordatotriloba* stems.


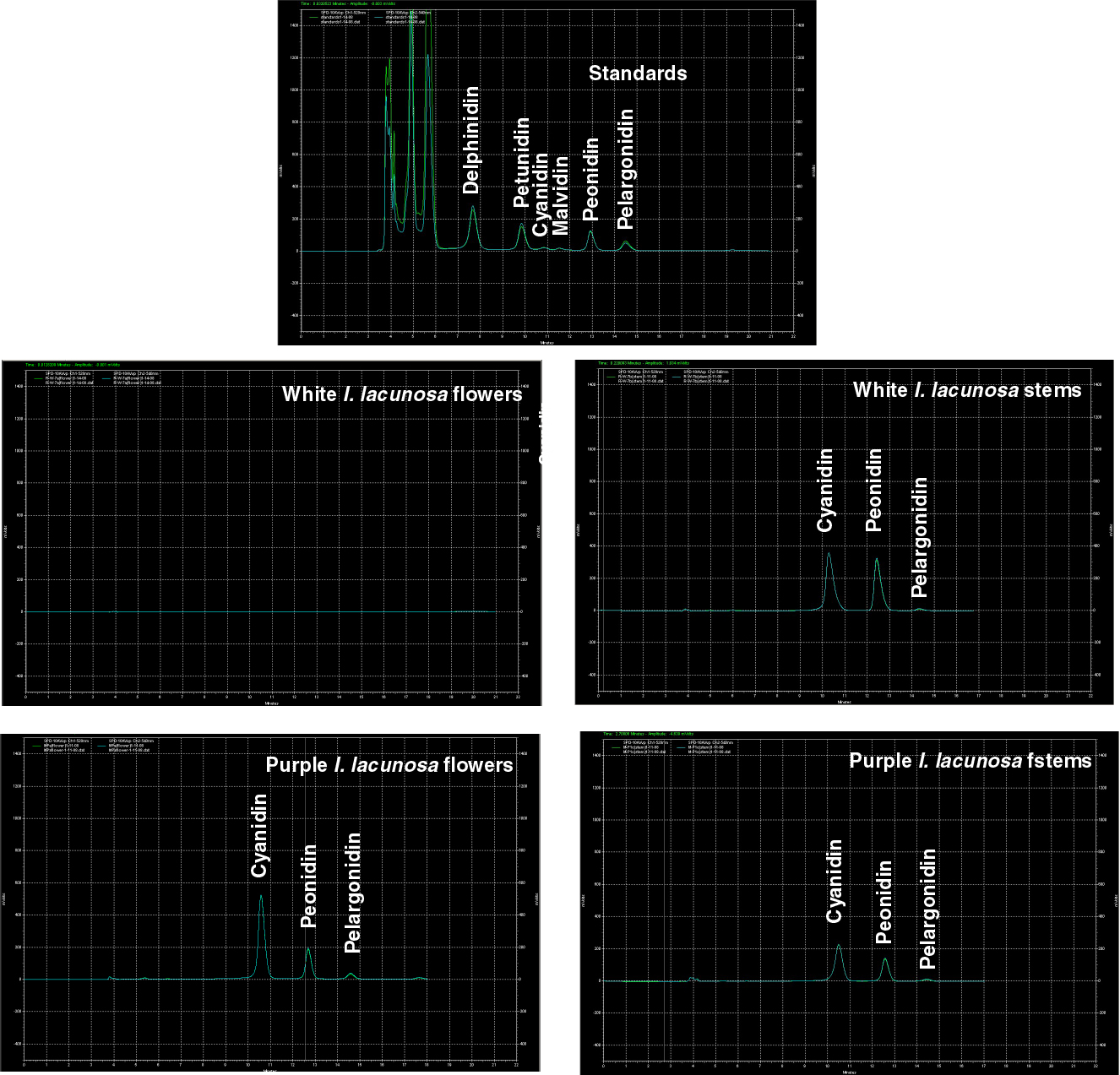

Supplement: S2 Fig — (DOCX) [file pone.0231263.s002.docx]
