# Supplementary material for: Selection favors loss of floral pigmentation in a highly selfing morning glory
Source: PLoS One. 2020 Apr 13;15(4):e0231263. doi: 10.1371/journal.pone.0231263 (PMC7153891; doi:10.1371/journal.pone.0231263)

Figure S3. Three phenotypes of S2 offspring produced by selfing F1 individuals from a cross of a white-flowered *I. lacunosa* to a purple-flowered *I. lacunosa.* A. F1 individual. B. Individual with white throats and little or no pigment in the corolla limbs. C. Individual with white corolla limbs with rays of purple and purple throats. D. Individual with purple pigment in both the throat and corolla limb.

A B


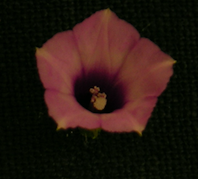

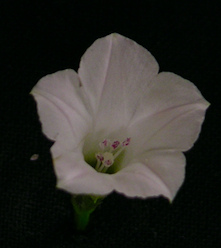


C D


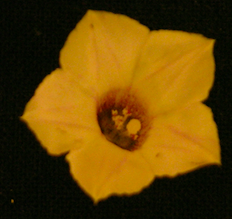

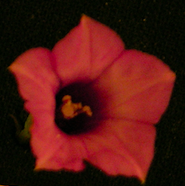

Supplement: S3 Fig — A. F1 individual. B. Individual with white throats and little or no pigment in the corolla limbs. C. Individual with white corolla limbs with rays of purple and purple throats. D. Individual with purple pigment in both the throat and corolla limb. (DOCX) [file pone.0231263.s003.docx]
