# Supplementary material for: Selection favors loss of floral pigmentation in a highly selfing morning glory
Source: PLoS One. 2020 Apr 13;15(4):e0231263. doi: 10.1371/journal.pone.0231263 (PMC7153891; doi:10.1371/journal.pone.0231263)
Supplement: S4 Fig — - - - - - - - indicates indel; ●●●●●●● indicates not sequenced. (DOCX) [file pone.0231263.s004.docx]

Figure S4. Sequences of genes referred to in main text aligned with highly similar BLAST hits from *Ipomoea* species. ------- indicates indel; ●●●●●●● indicates not sequenced.

**A.**  **Chalcone synthase D.** Top *I. nil* Genbank Accession AB001818; bottom *I. lacunosa*. Red indicates nucleotide differences.

ATGGTGACCGTCGAGGAGGTCAGAAAGGCGCAACGCGCCGAGGGTCCGGCGACCATTCTG

●●●●●●●●CGTCGAGGAGGTCAGAAAGGCGCAACGCGCGGAGGGTCCGGCGACCATTCTG

GCCATCGGAACGGCCACGCCGGCCAACTGCGTCGACCAAAGCACCTACCCCGACTACTAT

GCCATCGGAACCGCCACGCCGGCCAACTGCGTCAACCAAAGCACCTACCCCGACTACTAT

TTTCGTATCACTAATAGCGA**C**CATA**TG**ACCGA**C**CTCAA**GC**A**A**AAATTTCAGCGCATGTGT

TTTCGTATCACTAATAGCGA**G**CATA**AA**ACCGA**G**CTCAA**AG**A**G**AAATTTCAGCGCATGTGT

GA**T**AAATCAATGATCAC**A**AAGAGATACATGCACTTAACCGAGGAAATATTGAAGGAAAAC

GA**C**AAATCAATGATCAC**G**AAGAGATACATGCACTTAACCGAGGAAATATTGAAGGAAAAC

CCAAGCTTTTGTGAATACATGGCGCCTTCTTTGGATGCCAGGCAAGACATTGTGGTGGTG

CCAAGCTTTTGTGAATACATGGCGCCTTC●●●●●●●●●●●●●●●●●●●●●●●●●●●●●●●

GAAGTCCCCAAACTTGGGAAAGAAGCCGCCCAGAGCGCCATCAAGGAGTGGGGCCAACCA

●●●●●●●●●●●●●●●●●●●●●●●●●●●●●●●●●●●●●●●●●●●●●●●●●●●●●●●●●●●●

**B. Dihydroflavonol-4 reductase.** Top *I. batatas* accession HQ441167.1; bottom *I. lacunosa*. Red indicates nucleotide differences.

ATGGTGGACGGTAATCATCCAAAAGTAGTGTGCGTCACCGGAGCTGCTGGCTTTATCGGC

●●●●●●●●●●●●●●●●●●●●●●●●●●●●●●●●●●●●●●●●●●●●●●●●●●●●●●ATCGGC

TCCTGGTTGGTCATGACACTCCTCCA**A**CGAGGCTACCATGTTCACGCCACCGTTCGAGAT

TCCTGGTTGGTCATGACACTCCTCCA**G**CGAGGCTACCATGTTCACGCCACCGTTCGAGAT

CCTGGGAACACAAA**G**AAGGTGAAACATCTGCTTGAACTACCGAAAGC**G**GACACGAATTTG

CCTGGGAACACAAA**A**AAGGTGAAACATCTGCTTGAACTACCGAAAGC**C**GACACGAATTTG

A**G**AGTATGGAAGGGGGTAATGGAGGAAGAAGGAAGCTTTGATGAAGCCATTGCAGGGTGT

A**C**AGTATGGAAGGGGGTAATGGAGGAAGAAGGAAGCTTTGATGAAGCCATTGCAGGGTGT

GAAGGTGTGTTTCATGTGGCCACCCCTATGGATTTCGATTCCAAGGATCCCGAGAATGAA

GAAGGTGTGTTTCATGTGGCCACCCCTATGGATTTCGATTCCAAGGATCCCGAGAATGAA

GTGATAAAACCA**G**CCATCAATGGAGTGCTCAACATTATAAACTCTTGCGT**C**AAAGCCAAA

GTGATAAAACCA**A**CCATCAATGGAGTGCTCAACATTATAAACTCTTGCGT**G**AAAGCCAAA

ACCGTGAAGAGGCTGGTTTTCACTTCCTCTGCCGGGACTCT**C**AACGTCCAACCA**C**AACAA

ACCGTGAAGAGGCTGGTTTTCACTTCCTCTGCCGGGACTCT**T**AACGTCCAACCA**A**AACAA

AAGCCT**G**TGTACGATGAGAGCTGCTGGAGTGATCTGGATTTCATATATGCCAAGAAAATG

AAGCCT**A**TGTACGATGAGAGCTGCTGGAGTGATCTGGATTTCATATATGCCAAGAAAATG

ACTGGATGGATGTATTTTGCATCCAAAATACTGGCAGAGAAGGAAGCATGGAAAG**C**AACA

ACTGGATGGATGTATTTTGCATCCAAAATACTGGCAGAGAAGGAAGCATGGAAAG**T**AACA

AAAGAGAAGAAAATTGATTTCATAAGCATCATACCACCACTAGTGGTTGGCCCATTCATC

AAAGAGAAGAAAATTGATTTCATAAGCATCATACCACCACTAGTGGTTGGCCCATTCATC

ACCCCAACATTCCCACCCAGCCTCATCACTGCACTCTCACTAATTACTGGGAACCAAGCT

ACCCCAACATTCCCACCCAGCCTCATCACTGCACTCTCACTAATTACTGGGAACCAAGCT

CACTACTCCATCATTAAGCAAGGGCAGTATGTGCATCTGGATGATCTCTGCGAAGCTCAC

CACTACTCCATCATTAAGCAAGGGCAGTATGTGCATCTGGATGATCTCTGCGAAGCTCAC

ATATTCTTGTATGA**G**CATCCCAAAGCAGAAGGAAGATTCATCTGCTCTTCTCACCATACA

ATATTCTTGTATGA**A**CATCCCAAAGCAGAAGGAAGATTCATCTGCTCTTCTCACCATACA

ACCATCCA**T**GGTTTAG**G**GGAGATGATCAGACAGAATTGGCCTGAATACTACATCCCTTCT

ACCATCCA**C**GGTTTAG**C**GGAGATGATCAGACAGAATTGGCCTGAATACTACATCCCTTCT

**C**AGTTTAAGGGTATT**G**AAAAGGACTTGCCTGTGGTTTATTTTTCATCCAAGAAGTTGCAA

**G**AGTTTAAGGGTATT**C**AAAAGGACTTGCCTGTGGTTTATTTTTCATCCAAGAAGTTGCAA

GATATGGGGTTCCAGTTCAAGTAC**T**CTCTAGAGGACATGTACAGAGGAGCTATAGAGACC

GATATGGGGTTCCAGTTCAAGTAC**A**CTCTAGAGGACATGTACAGAGGAGCTATAGAGACC

TT**A**AGGAAGAA**G**GGGTTACTTCCCTATTCTACTAAAGA**GC**CTGCTGCAATT**G**A**A**GAAGA-

TT**G**AGGAAGAA**A**GGGTTACTTCCCTATTCTACTAAAGA**AG**CTGCTGCAATT**C**A**T**GAAGA**A**

--GCAAGAGACAGTGCCCTTAAAAGTCCAAGAACCTACTAAGCAAGAGGCCACAACAGTG

**GA**GCAAGAGACAGTGCCCTTAAAAGTCCAAGAACCTACTAAGCAAGAGGCCAC●●●●●●●

CCCTTAAAACCTGCTGCCATTGAACAAAAGCAAGAGACTGTGCCCTTAAAATTGGAAGAA

●●●●●●●●●●●●●●●●●●●●●●●●●●●●●●●●●●●●●●●●●●●●●●●●●●●●●●●●●●●●

CCCACTGCCATTGAACAAAAGCAAAAGGTAGTGCCCTTAAAAGCTTGA

**●●●●●●●●●●●●●●●●●●●●●●●●●●●●●●●●●●●●●●●●●●●●●●●●**

**C. R2R3-Myb Coding Region.** Top *I. purpurea* accession EU032617; bottom *I. lacunosa*. Red indicates nucleotide differences.

ATGGTTAATTCTTCTGCAAGGT**G**GTCTCCGCGAGTGAGAAA**G**GGTGC**T**TGGTCGGAAGAA

●●●●●●●●●●●●●●●●●●●●●T**C**GTCTCCGCGAGTGAGAAA**A**GGTGC**A**TGGTCGGAAGAA

GAAGATGATCTTTTGAGGAAGTGCATTCAGAAATTTGGTGAAGGAAAATGGCACCTAGTT

GAAGATGATCTTTTGAGGAAGTGCATTCAGAAATTTGGTGAAGGAAAATGGCACCTAGTT

CC**C**TTTAGAGCTGGGTTGAATAGGTGCAGAAAAAGTTGCAGATTGAGATGGTTGAACTAT

CC**A**TTTAGAGCTGGGTTGAATAGGTGCAGAAAAAGTTGCAGATTGAGATGGTTGAACTAT

CTCCATCCTGATATAAAGAGAGGCCATTTCAGTTTGGAAGAAGCTGATCTCATTCT**A**CGC

CTCCATCCTGATATAAAGAGAGGCCATTTCAGTTTGGAAGAAGCTGATCTCATTCT**G**CGC

CTCCATAAGCTCTTAGGCAACAGGTGGTCGCTTATTGCTGGCAGAATTCCGGGACGAACA

CTCCATAAGCTCTTAGGCAACAGGTGGTCGCTTATTGCTGGCAGAATTCCGGGACGAACA

GCAAACGATGTGAAGAATTACTGGCACAGCCATCTTAAGAAGAAGGTAGTT**A**GCATGCAT

GCAAACGATGTGAAGAATTACTGGCACAGCCATCTTAAGAAGAAGGTAGTT**G**GCATGCAT

ATG---**G**CTTC**A**TCTAATAGCAGCAGGCAAGATAATAATTGGGATGATGAGAAG**G**GCAAA

ATGACA**A**CTTC**T**TCTAATAGCAGCAGGCAAGATAATAATTGGGATGATGAGAAG**A**GCAAA

GCCCCACAAATCA**AG**GAAAACA**T**CCTCTTTAGGCCTCGACCTAGGAGATTCTTTAGGACC

GCCCCACAAATCA**CA**GAAAACA**C**CCTCTTTAGGCCTCGACCTAGGAGATTCTTTAGGAC-

TCGTTGTCATCTCC**G**GCGTTGTCGACATTAACCGGAAAAGCTAAGGCTGTCGTCTATGAT

--------ATCTCC**T**GCGTTGTCGACATTAACCGGAAAAGCT------------------

GCTCCTCCTCCTCCTCCTCCTCATCAACTCCAA**C**CA**C**AGC**C**G**GAA**G**C**A**A**C**GT**CGCC**CG**CG

---------CCTCCTCCTCCTCATCAACTCCAA**G**CA**T**CGC**A**G**TCG**G**A**A**G**C**AA**CGCC**GC**CG

**G**CGGACTTGCTAATGGTA**TT**TAATGTCCAACAAAA**C**A**G**TAACTCAATCGCGACTAATTTG

**C**CGGACTTGCTAATGGTA**AA**TAATGTCCAACAAAA**T**A**A**TAACTCAATCGCGACTAATTTG

CC**GG**CA**C**AAACAACG**G**CGCCGTCGTCCCACGACGGCGTG**A**AGTGGTGGGAAGATTTGCTC

CC**AT**CA**G**AAACAACG**T**CC------------------GTG**C**AGTGGTGGGAAGATTTGCTC

TACGACGA**T**A**G**T**C**A**C**CAAGGACTAATTGATTGGACGACTGATGATGACTTTCCGATTGAT

TACGACGA**C**A**A**T**G**A**A**CA●●●●●●●●●●●●●●●●●●●●●●●●●●●●●●●●●●●●●●●●●●●

GTGGACCTTTTAAAACTTTTAGACACAACCATTTAA

●●●●●●●●●●●●●●●●●●●●●●●●●●●●●●●●●●●●

**D. Partial *Dfr-B* sequence used in co-segregation assay.** Top: *I. lacunosa*; bottom: *I.* X *leucantha.* Green: NdeI restriction enzyme cut site.

GTCTAGCCATGTCCGTAGTATAAACCATATAGACTAGCCCTATCCATTATAGCAAATGGG

GTCTAGCCATGTCCGTAGTATAAACCAAATAGACTAGCCCTATCCATTATAGCAAATGGG

CTGATTGAGCCAAGCCGATCCAAATTGATCAGACTTGGACTTAATTTTAATTTCAGGTAG

CTGATTGAGCCAAGCCGATCCAAATTGATCAGACTTGGACTTAATTTTAATTTCA**GCTAG**

CCTGCTTGTTTGACACCTCTACTAATAATCAACCACAATCCCCCATCAAGCTAGGTGGCT

**C**CTGTTTGTTTGACACCTCTACTAATAATCAACCACAATCCCCAATCAAGCTAGGTGGCT

TGTAATATAAGAATGCCATTTGTTGGTTATTTTGATAGGTTTAAGGGTATTCAAAAGGAC

TGTAATATAAGAATGCCATTTGTTGGTTATTTTGATAGGTTTAAGGGTATTCAAAAGGAC

TTGCCTGTGGTTTATTTTTCATCCAAGAAGTTGCAAGATATGGGGTTCCAGTTCAAGTAC

TTGCCTGTGGTTTATTTTTCATCCAAGAAGTTGCAAGATATGGGGTTCCAGTTCAAGTAC

ACTCTAGAGGACATGTACAGAGGAGCTATAGAGACCTTGAGGAAGAAAGGGTTACTTCCC

TCTCTAGAGGACATGTACAGAGGAGCTATAGAGACCTTGAGGAAGAAAGGGTTACTTCCC

TATTCTACTAAAGAAGCTGCTGCAATTCATGAAGAAGAGCAAGAGACAGTGCCCTTAAAA

TATTCTACTAAAGAAGCTGCTGCAATTCATGAAGAAGAGCAAGAGACAGTGCCCTTAAAA

GTCCAAGAACCTACTAAGCAAGAGGCCACAACA

GTCCAAGAACCTACTAAGCAAGAGGCCACAACA

**E. R2R3-Myb partial coding region plus 3’ region**. Top *I. lacunosa*; bottom *I.* X *leucantha*. Red indicates nucleotide differences. Green: AseI restriction enzyme cut site; Blue: Stop codon.

GTCGCTTATTGCTGGCAGAATTCCGGGACGAACAGCAAACGATGTGAAGAATTACTGGCA

GTCGCTTATTGCTGGCAGAATTCCGGGACGAACAGCAAACGATGTGAAGAATTACTGGCA

CAGCCATCTTAAGAAGAAGGTAGTTGGCATGCATATGACAACTTCTTCTAATAGCAGCAG

CAGCCATCTTAAGAAGAAGGTAGTTGGCATGCATATGACAACTTCTTCTAATAGCAGCAG

GCAAGATAATAATTGGGATGATGAGAAGAGCAAAGCCCCACAAATCACAGAAAACACCCT

GCAAGATAATAATTGGGATGATGAGAAGAGCAAAGCCCCACAAATCACAGAAAACACCCT

CTTTAGGCCTCGACCTAGGAGATTCTTTAGGACATCTCCTGCGTTGTCGACATTAACCGG

CTTTAGGCCTCGACCTAGGAGATTCTTTAGGACATCTCCTGCGTTGTCGACATTAACCGG

AAAAGCTCCTCCTCCTCCTCATCAACTCCAAGCATCGCAGTCGGAAGCAACGCCGCCGCC

AAAAGCTCCTCCTCCTCCTCATCAACTCCAAGCATCGCAGTCGGAATCAACGCCGCCGCC

GGACTTGCTAATGGTAAATAATGTCCAACAAAATAATAACTCAATCGCGACTAATTTGCC

GGACTTGCTAATGGTAAATAATGTCCAACAAAATAATAACTCAATCGCGACTAATTTGCC

ATCAGAAACAACGTCCGTGCAGTGGTGGGAAGATTTGCTCTACGACGACAATGAACA---

ATCAGAAACAACGTCCGTGCAGTGGTGGGAAGATTTGCTCTACGACGACAATGAACAACT

------CCAAGGAACCACCGACATGCACCAAGGAATAATCGATTGGACTGATGATGGCTT

CAATCACCAAGGAACCACCGACATGCACCAAGGAATAATCGATTGGACTGATGATGGCTT

TCCGATTGATGTGGACCTTTTAACACTTTTAGACCCAACAAAC**TAA**CATGGAATCCTTAA

TCCGATTGATGTGGACCTTTTAACACTTTTAGACCCAACAAAC**TAA**CATGGAATCCTTAA

ATCAACCGTATCATGTCACTATATATTTCTTTGTATTTTATTAAGACCGTCTACACCTAT

ATCAACCGTATCATGTCACTATATATTTCTTTGTATTTTATTAAGACCG-----------

GTAATCTTCTCTCACTATCAAAATATCATATT**ATTAAT**CATTATTCATTCTTCATACAAT

--------------------------------------------TCATTCTTCATACAAT

TCTCTTTTCTCTTTCAGAAATGATCAACTTTTATATAGCTTTATACAAAATTATTTCTTC

TCTCTTTTCTCTTTCAAAAATGATCAACTTTTATATAGCTTTATACAAAATTA-TTCTTC

AATCAATAAAAAAATACTAATAATAATAATTAAAAATTTGAAA**C**GTGCCTTTGCAATTTT

AATCAATAAAAAAATACTAATAATAATAATTAAAAATTTGAAA**A**GTGCCTTTGCAATTTT

GACAATAGAATTGTAAATTTGCTTTAGTCAGAATTGCAAAAGCTAAAGACGTGGCAATTT

GACAATAGAATTGTAAATTTGCTTTAGTCAGAATTGCAAAAGCTAAAGACGTGGCAATTT
