# Supplementary material for: Selection favors loss of floral pigmentation in a highly selfing morning glory
Source: PLoS One. 2020 Apr 13;15(4):e0231263. doi: 10.1371/journal.pone.0231263 (PMC7153891; doi:10.1371/journal.pone.0231263)
Supplement: S1 Table — DFR-B and CHS-D as well as the anthocyanin transcription factor R2R3-Myb. (DOCX) [file pone.0231263.s005.docx]

Table S1: Primers used to amplify partial regions of anthocyanin genes

*DFR-B* and *CHS-D* as well as the anthocyanin transcription factor *R2R3-Myb*.

|  | Forward Primer (5'→3') | Reverse Primer (5'→3') |
| --- | --- | --- |
| *DFR-B* (cDNA) | ATCGGCTCCTGGTTGGTCATGACA | GTGGCCTCTTGCTTAGTAGGTTCTT |
| *CHS-D* (cDNA) | CGTCGAGGAGGTCAGAAAGG | TGGAGGACCACGGTTTCGATGGTAA |
| *R2R3-Myb* (cDNA) | TCGTCTCCGCGAGTGAGAA | TGTTCATTGTCGTCGTAGAGCAAAT |
| *R2R3-Myb* (3’ UTR genomic region) | TCGTCTCCGCGAGTGAGAA | CAATTTGCAGTGATGCCAAG |
