# Supplementary material for: Selection favors loss of floral pigmentation in a highly selfing morning glory
Source: PLoS One. 2020 Apr 13;15(4):e0231263. doi: 10.1371/journal.pone.0231263 (PMC7153891; doi:10.1371/journal.pone.0231263)
Supplement: S2 Table — (DOCX) [file pone.0231263.s006.docx]

Table S2: Primers used for Q-PCR expression assay.

|  | Forward Primer (5'→3') | Reverse Primer (5'→3') |
| --- | --- | --- |
| *CHS-D* | GCACCTACCCCGACTACTATTTTCGT | GAAGGCGCCATGTATTCACAAAAGC |
| *DFR-B* | ATCGGCTCCTGGTTGGTCAAGACAC | GCAATGGCTTCATCAAAGCTTCCTT |
| *R2R3-Myb* | TCGACATTAACCGGAAAAGC | AAATCTTCCCACCACTGCAC |
| *EF-1** | CTGTAACAAGATGGATGCCA | AGACGGAGTGGCTTGTCTG |
|  | * primers developed in Streisfeld and Rausher, 2009 ([Streisfeld & Rausher, 2009](#_ENREF_110)) | |
