# Supplementary material for: Selection favors loss of floral pigmentation in a highly selfing morning glory
Source: PLoS One. 2020 Apr 13;15(4):e0231263. doi: 10.1371/journal.pone.0231263 (PMC7153891; doi:10.1371/journal.pone.0231263)
Supplement: S3 Table — (DOCX) [file pone.0231263.s007.docx]

Table S3: Primers and restriction enzymes used in co-segregation assay.

| Gene | Genetic Variation (cut site bold) | Rest.  Enz. | Forward Primer (5'→3') | | Reverse Primer (5'→3') | PCR &  Restrict. Frag. size (b.p) | |  |
| --- | --- | --- | --- | --- | --- | --- | --- | --- |
| ***DFR-B*** |  |  |  |  | |  | |  |
|  |  |  |  |  | |  | |  |
| *I. X leucantha* | TTTCA**GCTAGC** | NheI | GTCGCTTATTGCTGGCAGAA | GTCTAGCCATGTCCGTAGTATAAACCAA | | | 120, 330 |  |
|  |  |  |  |  | |  | |  |
|  |  |  |  |  | |  | |  |
| *I. lacunosa* | TTTCAGGTAGC |  |  |  | | 450 | |  |
|  |  |  |  |  | |  | |  |
| ***R2R3-Myb*** |  |  |  |  | |  | |  |
|  |  |  |  |  | |  | |  |
|  |  |  |  |  | |  | |  |
| *I. X leucantha* | ------------ | AseI | GTCTAGCCATGTCCGTAGTATAACCAA | CAATTTGCAGTGATGCCAAGA | | 890 | |  |
| *I. lacunosa* | TATT**ATTAAT**TC |  |  |  | | 630, 260 | |  |
|  |  |  |  |  | |  | |  |
