# Supplementary material for: Selection favors loss of floral pigmentation in a highly selfing morning glory
Source: PLoS One. 2020 Apr 13;15(4):e0231263. doi: 10.1371/journal.pone.0231263 (PMC7153891; doi:10.1371/journal.pone.0231263)
Supplement: S4 Table — 100 flowers were counted when populations were fixed for flower color. 200 flowers were counted when variation in flower color was found. (DOCX) [file pone.0231263.s008.docx]

Table S4: Frequency of white allele in censused populations of *I. cordatotriloba* and *I. lacunosa.* 100 flowers were counted when populations were fixed for flower color. 200 flowers were counted when variation in flower color was found.

| # pop | Population | latitude (N) | longitude (W) | Species | frequency of white allele |
| --- | --- | --- | --- | --- | --- |
| 1 | c14 | 34.10871 | 78.10811 | *I. cordatotriloba* | 0 |
| 2 | c9 | 33.92639 | 80.15923 | *I. cordatotriloba* | 0 |
| 3 | c6 | 33.87177 | 78.62401 | *I. cordatotriloba* | 0 |
| 4 | c11 | 33.94935 | 79.02579 | *I. cordatotriloba* | 0 |
| 5 | c2 | 33.73008 | 78.90894 | *I. cordatotriloba* | 0 |
| 6 | c8 | 33.90598 | 78.58437 | *I. cordatotriloba* | 0 |
| 7 | c10 | 33.93110 | 78.55347 | *I. cordatotriloba* | 0 |
| 8 | c13 | 34.00996 | 78.30176 | *I. cordatotriloba* | 0 |
| 9 | c16 | 34.19313 | 78.06443 | *I. cordatotriloba* | 0 |
| 10 | c17 | 34.22374 | 78.01118 | *I. cordatotriloba* | 0 |
| 11 | c18 | 34.26725 | 78.09312 | *I. cordatotriloba* | 0 |
| 12 | c19 | 34.30962 | 77.97134 | *I. cordatotriloba* | 0 |
| 13 | c20 | 34.34259 | 78.00364 | *I. cordatotriloba* | 0 |
| 14 | c21 | 34.35167 | 77.72792 | *I. cordatotriloba* | 0 |
| 15 | c23 | 34.39078 | 77.67836 | *I. cordatotriloba* | 0 |
| 16 | c24 | 34.48462 | 77.56372 | *I. cordatotriloba* | 0 |
| 17 | c26 | 34.60050 | 77.94480 | *I. cordatotriloba* | 0 |
| 18 | c28 | 34.64724 | 77.96371 | *I. cordatotriloba* | 0 |
| 19 | cla2 | 34.42570 | 77.87385 | *I. cordatotriloba* | 0 |
| 20 | cu1 | 34.57249 | 77.94509 | *I. cordatotriloba* | 0 |
| 21 | c40 | 35.09979 | 77.05437 | *I. cordatotriloba* | 0 |
| 22 | c39 | 35.08108 | 77.03062 | *I. cordatotriloba* | 0 |
| 23 | c38 | 35.02278 | 76.99767 | *I. cordatotriloba* | 0 |
| 24 | c37 | 34.96248 | 76.95459 | *I. cordatotriloba* | 0 |
| 25 | c35 | 34.86991 | 76.90060 | *I. cordatotriloba* | 0 |
| 26 | c34 | 34.85374 | 76.89292 | *I. cordatotriloba* | 0 |
| 27 | c32 | 34.83395 | 76.87960 | *I. cordatotriloba* | 0 |
| 28 | c25 | 34.51701 | 77.91437 | *I. cordatotriloba* | 0 |
| 29 | clela4 | 34.41868 | 77.88434 | *I. cordatotriloba* | 0 |
| 30 | cla1 | 33.90135 | 79.13961 | *I. cordatotriloba* | 0 |
| 31 | clela2 | 33.95099 | 79.05856 | *I. cordatotriloba* | 0 |
| 32 | clela3 | 33.95991 | 78.99147 | *I. cordatotriloba* | 0 |
| 33 | cula2 | 34.00710 | 79.22410 | *I. cordatotriloba* | 0 |
| 34 | c7 | 33.89953 | 80.89953 | *I. cordatotriloba* | 0.04 |
| 35 | c3 | 33.74665 | 78.83166 | *I. cordatotriloba* | 0.10 |
| 36 | c22 | 34.37833 | 77.89637 | *I. cordatotriloba* | 0.11 |
| 37 | c15 | 34.18635 | 78.07789 | *I. cordatotriloba* | 0.05 |
| 38 | c27 | 34.63702 | 77.92169 | *I. cordatotriloba* | 0.18 |
| 39 | clula1 | 33.93341 | 79.10781 | *I. cordatotriloba* | 0.47 |
| 40 | cle1 | 33.85097 | 78.81234 | *I. cordatotriloba* | 0.23 |
| 41 | cl4 | 34.81235 | 76.87686 | *I. cordatotriloba* | 0.43 |
| 42 | c1 | 33.71054 | 78.88154 | *I. cordatotriloba* | 1 |
| 43 | c4 | 33.75048 | 78.97870 | *I. cordatotriloba* | 1 |
| 44 | c5 | 33.86581 | 78.63960 | *I. cordatotriloba* | 1 |
| 45 | c12 | 33.96198 | 78.42105 | *I. cordatotriloba* | 1 |
| 46 | c36 | 34.95646 | 76.95004 | *I. cordatotriloba* | 1 |
| 47 | c33 | 34.85135 | 76.88751 | *I. cordatotriloba* | 1 |
| 48 | c29 | 34.69087 | 77.97886 | *I. cordatotriloba* | 1 |
| 49 | c30 | 34.69795 | 76.78481 | *I. cordatotriloba* | 1 |
| 50 | c31 | 34.74557 | 77.97576 | *I. cordatotriloba* | 1 |
| 51 | ula3 | 34.52426 | 79.27620 | *I. lacunosa* | 1 |
| 52 | ula4 | 34.52973 | 79.11169 | *I. lacunosa* | 1 |
| 53 | la9 | 34.69638 | 77.97226 | *I. lacunosa* | 1 |
| 54 | la10 | 34.74071 | 77.97177 | *I. lacunosa* | 1 |
| 55 | ula3 | 34.75478 | 76.84532 | *I. lacunosa* | 1 |
| 56 | ula5 | 34.76182 | 77.97152 | *I. lacunosa* | 0.44 |
| 57 | la11 | 34.79800 | 79.88852 | *I. lacunosa* | 1 |
| 58 | ul4 | 34.81235 | 76.87686 | *I. lacunosa* | 1 |
| 59 | ula6 | 34.83951 | 78.95704 | *I. lacunosa* | 1 |
| 60 | ula7 | 34.87798 | 77.97215 | *I. lacunosa* | 1 |
| 61 | la12 | 34.89755 | 79.80206 | *I. lacunosa* | 1 |
| 62 | la13 | 34.90111 | 78.22715 | *I. lacunosa* | 1 |
| 63 | ula8 | 34.90256 | 77.99157 | *I. lacunosa* | 1 |
| 64 | ula9 | 34.92800 | 78.03465 | *I. lacunosa* | 1 |
| 65 | la14 | 34.96051 | 78.92429 | *I. lacunosa* | 1 |
| 66 | la15 | 34.95980 | 78.17761 | *I. lacunosa* | 1 |
| 67 | la17 | 35.16163 | 78.72521 | *I. lacunosa* | 1 |
| 68 | la18 | 35.23980 | 77.57603 | *I. lacunosa* | 1 |
| 69 | la19 | 35.24480 | 78.33661 | *I. lacunosa* | 1 |
| 70 | la20 | 35.27125 | 78.61682 | *I. lacunosa* | 1 |
| 71 | la21 | 35.28423 | 77.79586 | *I. lacunosa* | 1 |
| 72 | la22 | 35.32614 | 79.28025 | *I. lacunosa* | 0.98 |
| 73 | la23 | 35.33331 | 79.36028 | *I. lacunosa* | 1 |
| 74 | la24 | 35.33467 | 79.36006 | *I. lacunosa* | 1 |
| 75 | la25 | 35.33599 | 79.35348 | *I. lacunosa* | 1 |
| 76 | la26 | 35.33956 | 78.55302 | *I. lacunosa* | 1 |
| 77 | la27 | 35.36075 | 77.92425 | *I. lacunosa* | 1 |
| 78 | la28 | 35.46023 | 78.54914 | *I. lacunosa* | 0.99 |
| 79 | la29 | 35.47045 | 78.19380 | *I. lacunosa* | 1 |
| 80 | la30 | 35.51501 | 79.34398 | *I. lacunosa* | 1 |
| 81 | la32 | 35.63225 | 79.01803 | *I. lacunosa* | 1 |
| 82 | la33 | 35.68255 | 78.91405 | *I. lacunosa* | 1 |
| 83 | la34 | 35.86155 | 80.18288 | *I. lacunosa* | 1 |
| 84 | la35 | 35.86958 | 80.03384 | *I. lacunosa* | 1 |
| 85 | la36 | 35.93086 | 79.23156 | *I. lacunosa* | 1 |
| 86 | la37 | 35.93593 | 79.19526 | *I. lacunosa* | 1 |
| 87 | la38 | 35.95078 | 79.92552 | *I. lacunosa* | 1 |
| 88 | la39 | 35.95091 | 79.25802 | *I. lacunosa* | 1 |
| 89 | la40 | 35.97611 | 79.25593 | *I. lacunosa* | 1 |
| 90 | la41 | 35.99383 | 78.94802 | *I. lacunosa* | 1 |
| 91 | la43 | 35.98721 | 79.26268 | *I. lacunosa* | 0.83 |
| 92 | la44 | 36.04138 | 79.55877 | *I. lacunosa* | 1 |
| 93 | la45 | 36.07438 | 79.46184 | *I. lacunosa* | 1 |
