# Supplementary material for: Selection favors loss of floral pigmentation in a highly selfing morning glory
Source: PLoS One. 2020 Apr 13;15(4):e0231263. doi: 10.1371/journal.pone.0231263 (PMC7153891; doi:10.1371/journal.pone.0231263)
Supplement: S5 Table — Primers were developed and PCR optimized by Hu et al (Hu et al., 2004). (DOCX) [file pone.0231263.s009.docx]

Table S5. Microsatellite primers. Primers were developed and PCR optimized by Hu et al ([Hu et al., 2004](#_ENREF_57)).

|  | Forward Primer (5'→3') | Reverse Primer (5'→3') | Size (bp) | Tm (°C) |
| --- | --- | --- | --- | --- |
| ITSSR 02 | AGGTCAAGGTGGTTTTGGTTCTG | TTGCCCTCCAACAAGCATTCCC | 157-178 | 60 |
| ITSSR04 | GCGTAACACATAAATTGGACTG | GGAAGTTTGAAAAGGTTAAGCC | 205-209 | 60 |
| ITSSR07 | CACCATACCCAATTTTTACAGATGC | GATTGAATGAATGATGCGG | 160-172 | 56 |
| ITSSR14 | CTCCATTCAAACAGCGTCTC | CGGGCAATTCAGTTGACTC | 133-206 | 56 |
|  |  |  |  |  |
