# Supplementary material for: Selection favors loss of floral pigmentation in a highly selfing morning glory
Source: PLoS One. 2020 Apr 13;15(4):e0231263. doi: 10.1371/journal.pone.0231263 (PMC7153891; doi:10.1371/journal.pone.0231263)
Supplement: S6 Table — (DOCX) [file pone.0231263.s010.docx]

Table S6: AMOVA for microsatellite variation.

| Source of Variation | d.f. | Sum of Squares | Variance Components | Percentage of Variation | P |
| --- | --- | --- | --- | --- | --- |
| Between *I. lacunosa* and *I. cordatotriloba* | 1 | 19.068 | 0.032 | 4.24 | 0.13 |
| Among populations within *I. lacunosa* and *I. cordatotriloba* | 13 | 128.003 | 0.35 | 45.10 | 0 |
| Among individuals within populations | 192 | 118.47 | 0.23 | 29.61 | 0 |
| Within individuals | 207 | 33.50 | 0.16 | 21.05 | 0 |
