# Supplementary material for: Selection favors loss of floral pigmentation in a highly selfing morning glory
Source: PLoS One. 2020 Apr 13;15(4):e0231263. doi: 10.1371/journal.pone.0231263 (PMC7153891; doi:10.1371/journal.pone.0231263)
Supplement: S7 Table — (DOCX) [file pone.0231263.s011.docx]

Table S7: Flower color frequency for 15 populations with microsatellite data.

|  | population | latitude (N) | longitude (W) | Species | White Allele Frequency |
| --- | --- | --- | --- | --- | --- |
| 1 | c13 | 34.00996 | 78.30176 | *I. cordatotriloba* | 0 |
| 2 | clela3 | 33.95991 | 78.99147 | *I. cordatotriloba* | 0 |
| 3 | c7 | 33.89953 | 80.89953 | *I. cordatotriloba* | 0.04 |
| 4 | c22 | 34.37833 | 77.89637 | *I. cordatotriloba* | 0.11 |
| 5 | cle1 | 33.85097 | 78.81234 | *I. cordatotriloba* | 0.23 |
| 6 | cl4 | 34.81235 | 76.87686 | *I. cordatotriloba* | 0.43 |
| 7 | c19 | 34.6909 | 77.97886 | *I. cordatotriloba* | 1 |
| 8 | la43 | 35.98721 | 79.26268 | *I. lacunosa* | 0.83 |
| 9 | la3 | 34.22187 | 80.40211 | *I. lacunosa* | 1 |
| 10 | lela8 | 33.98603 | 80.17159 | *I. lacunosa* | 1 |
| 11 | la15 | 34.9598 | 78.17761 | *I. lacunosa* | 1 |
| 12 | la30 | 35.51501 | 79.34398 | *I. lacunosa* | 1 |
| 13 | la35 | 35.86958 | 80.03384 | *I. lacunosa* | 1 |
| 14 | la7 | 34.47367 | 79.8702 | *I. lacunosa* | 1 |
| 15 | ula7 | 34.87798 | 77.97215 | *I. lacunosa* | 1 |
